# Endotheliitis bei COVID-19

**DOI:** 10.1007/s00292-020-00875-9

**Published:** 2020-12-11

**Authors:** Zsuzsanna Varga

**Affiliations:** Institut für Pathologie und Molekularpathologie, Universitätsspital Zürich, Schmelzbergstraße 12, CH-8091 Zürich, Schweiz

**Keywords:** COVID-19, SARS-CoV-2, Endotheliitis, Diffuser Organbefall, COVID-19, SARS-CoV-2, Endotheliitis, Diffuse organ affection

## Abstract

Bei COVID-19-Infektion liegt eine systemische virale Reaktion gefolgt von einer entzündlichen Krankheitsphase vor. Die erste Phase verläuft meist mild/asymptomatisch. Nur ein Teil der Patienten entwickelt die entzündliche Phase mit hoher Mortalität. Patienten mit vorbestehenden kardiovaskulären Erkrankungen und kardiovaskulären Risikofaktoren haben ein höheres Risiko, schwer an COVID-19 zu erkranken. COVID-19 betrifft nicht nur das Lungenparenchym durch die ACE2-Rezeptoren. COVID-19 betrifft nicht nur das Lungenparenchym durch die ACE2-Rezeptoren, sondern kann auch im Gesamtkörper eine generalisierte endotheliale Schädigung und Entzündung im Sinne einer sog. Endotheliitis zur Folge haben. Die Morphologie der Endotheliitis stellt eine Akkumulation von Lymphozyten, Plasmazellen und Makrophagen im und unterhalb der endothelialen Zellen dar. Eine Endotheliitis kann eine Vasokonstriktion mit konsekutiver Organischämie, Entzündung und Gewebeödem sowie thrombotische Mikrozirkulationsstörung zur Folge haben. Patienten mit vorbestehender kardiovaskulärer Dysfunktion (Hypertonus, Diabetes mellitus, Übergewicht und weitere kardiovaskuläre Erkrankungen, männliches Geschlecht) haben ein erhöhtes Risiko für schwere Verläufe einer COVID-19-Infektion. Insbesondere endothelstabilisierende Ansätze kommen aus diesen Gründen zum Einsatz. Die Erkenntnisse wurden seit dem Pandemieausbruch mehrheitlich von Autopsien gewonnen.

## Abstract (en)

COVID-19 disease is a systemic inflammatory viral reaction starting with the viral phase followed by the inflammatory phase. The first phase is rather mild and asymptomatic with only a small subset of infected patients turning into the inflammatory phase with high mortality. Patients with pre-existing cardiovascular diseases and cardiovascular risk factors pose a considerably higher risk to develop severe or lethal COVID-19 disease course. COVID-19 affects not only the epithelial cells of the lung parenchyma via ACE2, but also endothelial cells across the whole body thus leading to generalized endothelial damage and inflammation, so-called endotheliitis. The histological morphology of endotheliitis comprises the accumulation of lymphocytes, plasma cells and macrophages beneath the endothelial cells and within the perivascular spaces. Endothelial cells play an important role in the regulation of vascular tone and the maintenance of vascular homeostasis. Endotheliitis thus can shift the vascular equilibrium towards more pronounced vasoconstriction with subsequent organ ischemia, inflammation with associated tissue edema and a procoagulant state. Patients with pre-existing endothelial dysfunction (male sex, smoking, hypertension, diabetes, obesity, and established cardiovascular disease) are particularly vulnerable and have adverse outcomes in COVID-19. This is a rationale for approaches to stabilize the endothelium. Most of these findings have been established from autopsies since the outbreak of the pandemic.

## Hintergrund und klinische Aspekte

Das Coronavirus SARS-CoV‑2 wurde Ende 2019 von ersten kranken Patienten aus Korea isoliert und aus den nasopharyngealen Abstrichpräparaten wurden erstmals labortechnisch mit Sars-CoV‑2 infizierte Zellen nachgewiesen [1]. Die viralen Strukturen in diesen ersten infizierten Zellkulturen zeigten einen klaren zytopathischen Effekt und haben sich als runde Partikel umgeben von einen prominenten äußeren Ring, welcher an eine Krone („crown-like spikes“) erinnert, erwiesen [1]. Die die Sars-Cov-2-Infektion (die sog. COVID-19-Erkrankung) begleitenden Krankheiten (Komorbiditäten) zeigen während der Pandemie keine geografische Spezifität, sondern präsentieren sich vielmehr unabhängig von den Kohorten und von den Kontinenten mit einem sehr ähnlichen klinischen Bild [2, 3]. Die Komorbiditäten sind vor allem in den schweren Verläufen vermehrt: chronische obstruktive Lungenerkrankungen (COPD), Diabetes mellitus, Hypertonus, koronare Herzkrankheit, zerebrovaskuläre Erkrankungen oder Tumorerkrankungen [2, 3]. Ein sehr ähnliches klinisches Bild konnte anhand der ersten größeren Kohorten aus New York und aus China charakterisiert werden [2, 3]. Interessanterweise waren chronische Nierenerkrankungen, Übergewicht, ein hoher BMI und das männliche Geschlecht bei COVID-19-Infektion vermehrt zu finden [2, 3].

## Vaskuläre Beteiligung und histologische Aspekte

**Figure 1:**
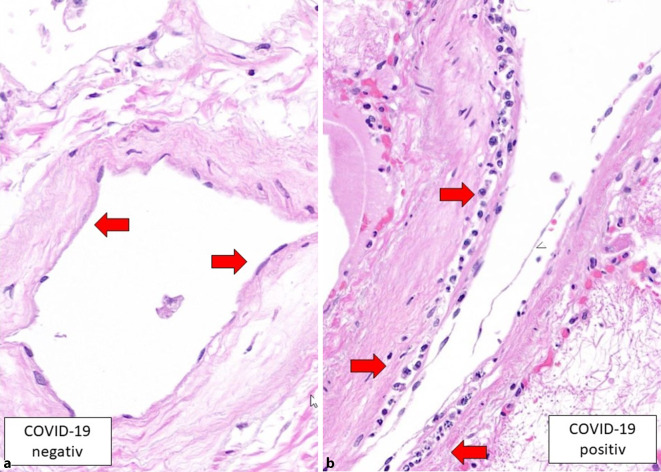

**Figure 2:**
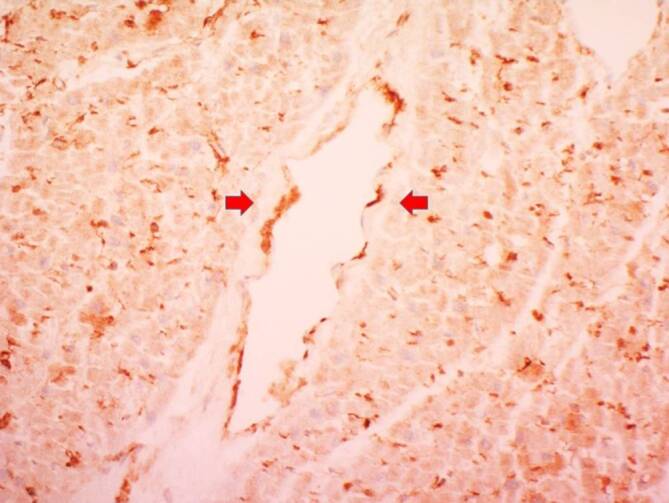

Es wurde bereits in den ersten Befunden nach COVID-19-Obduktionen beobachtet, dass diverse Organe pathologische Veränderungen in den Gefäßen zeigten [4], nicht nur in den pulmonalen Gefäßen, wo die Erkrankung sich klinisch mit den schwersten Symptomen präsentiert, sondern auch in den submukosalen Darmgefäßen, in der Herzmuskulatur oder auch in der Leber [4]. Diese Morphologie hat sich in Form einer sog. Endotheliitis präsentiert, welche im Grunde eine Akkumulation von Lymphozyten, Plasmazellen und Makrophagen im und unterhalb der endothelialen Zellen zur Folge hat. Diese gemischten Entzündungszellen haben mehrheitlich die Arteriolen/Venolen befallen, waren aber auch in den Kapillaren und ab und zu in den mittelgroßen Gefäßen in den genannten Organen nachweisbar (Abb. 1). Das SARS-CoV-2-Virus kann durch die sog. ACE2(„angiotensin coverting enzyme 2“)- und die TMPRSS(„transmembrane protease serine 2“)-Rezeptoren in die Zelle gelangen und sie infizieren. Wie es in Abb. 2 am Bespiel des Myokards dargestellt wird, sind ACE2-Rezeptoren in den kleinkalibrigen intramyokardialen Gefäßen, welche den Eintritt des Virus in die Zelle ermöglichen, reichlich vorhanden (Abb. 2). Ein weiterer Aspekt der Zellinfiltrate ist dadurch charakterisiert, dass die Entzündungszellen eine vermehrte apoptotische Aktivität zeigten, welche eine Art intraendothelialen apoptotischen Zelluntergang zur Folge hatten [4]. In den immunhistochemischen Reaktionen für Caspase 3 konnte die gesteigerte apoptotische Aktivität der Entzündungszellen und der benachbarten weiteren Zellelemente (wie Endothel, Kryptenepithel, Hepatozyten usw.) immunhistochemisch belegt werden [4]. Aufgrund der ersten Autopsiebefunde konnten 3 Mechanismen identifiziert werden, welche zu COVID-19-assoziierten vaskulären Gewebeschädigung und Mikrozirkulationsstörung führten [4]. Zum einen liegt eine disseminierte Endotheliitis vor, welche diverse Organe wie Lunge, Dünndarm, Niere, Myokard und Leber befallen kann, zum anderen ist eine disseminierte thrombotische Mikroangiopathie der kleinen Gefäße zu beobachten, welche durch Fibrinthromben und leukozytäre Thromben charakterisiert ist und welche bei schweren Verläufen von Dünndarmischämien zugrunde liegt. Als ein weiterer Aspekt der Gefäßschädigung konnte eine gesteigerte apoptotische Aktivität sowohl in der endothelialen entzündlichen Reaktion als auch im benachbarten Endothel/Epithel nachgewiesen werden [4].

## Vaskuläre Beteiligung und molekulare Aspekte

Die durch SARS-CoV‑2 verursachten Gefäßschädigungen sind auf einen komplexen Pathomechanismus zurückzuführen [7, 8]. Die Mikrozirkulationsstörung, welche durch den Zelltod der beteiligten Zellen den ersten pathomechanistischen Schritt darstellt, wird durch Faktoren wie IL-1-beta, TNF usw. begünstigt, welche eine Störung der interendothelialen Junktionen zur Folge haben [7, 8]. Die Aktivierung von weiteren Zytokinen wie IL‑6, IL‑8, Bradykinine, VEGF bedingt eine erhöhte intrazelluläre Kontraktilität [8]. Die gesteigerte Koagulation wird durch die Aktivierung der Koagulationskaskade durch P‑Selektin, Von-Willebrand-Faktor und durch weitere Faktoren wie GM-CSF begünstigt [8]. Die entzündliche Begleitreaktion wird durch die Aktivierung der Komplementkaskade und durch leukozytäre Adhäsionsmoleküle wie VCAM1, E‑Selectin, ICAM1 reguliert [8].

Die erste molekulare Evidenz, dass SARS-CoV‑2 extrapulmonäre Strukturen, wie z. B. Nierengewebe, infizieren kann, wurde in vaskulären kapillären Organoiden bereits gezeigt [9]. Montelli et al. haben SARS-CoV-2-virale RNA in CD31-positiven Nierenorganoiden nach einer SARS-CoV-2-Infektion nachgewiesen, welche den ersten Beweis dafür lieferte, dass SARS-CoV‑2 die Fähigkeit hat, direkt ins Endothel eines extrapulmonalen Organs einzugreifen [9]. Ein RNA-basierter Multiorganbefall durch SARS-CoV‑2 wurde anschließend in einer größeren Autopsiestudie nachgewiesen. Zusätzlich zu den bereits beschriebenen Organen wie Lunge, Trachea, Nieren und Leber wurde RNA auch im Hirn und im Blut nachgewiesen, was den Multiorgantropismus von SARS-CoV‑2 weiter belegt [10, 11].

## Klinische Relevanz

Bei SARS-CoV-2-Infektion ist eine virale Reaktion gefolgt von einer entzündlichen Reaktion zu beobachten [2, 3]. Die erste Phase verläuft meist mild/asymptomatisch, nur ein Teil der Patienten und Patientinnen entwickelt die entzündliche Phase mit hoher Mortalität [2, 3]. COVID-19 betrifft nicht nur das Lungenparenchym durch ACE2 und TMPRSS [4–6]. Der Multiorganbefall durch SARS-CoV‑2 induziert eine generalisierte Endothelschädigung und endotheliale Entzündung im Sinne einer Endotheliitis [4]. Die Endotheliitis kann eine Vasokonstriktion mit konsekutiver Organischämie, Entzündung und Gewebeödem sowie thrombotische Mikrozirkulationsstörungen zur Folge haben [4–6]. Bei Patienten und Patientinnen mit bereits vorhandener kardiovaskulärer Dysfunktion (Hypertonus, Diabetes mellitus, Übergewicht und weitere kardiovaskuläre Erkrankungen) sowie männlichen Geschlechts besteht ein besonders hohes Risiko für schwere Verläufe einer COVID-19-Infektion [2, 3]. Endothelstabilisierende Therapien kommen aus diesen Gründen bei COVID-19-Erkrankung besonders zum Einsatz [4, 5].

Die ersten Erkenntnisse der COVID-19-Pathomechanismen wurden mehrheitlich durch Autopsien gewonnen.
